# Treatment Compliance with Fixed-Dose Combination of Vildagliptin/Metformin in Patients with Type 2 Diabetes Mellitus Inadequately Controlled with Metformin Monotherapy: A 24-Week Observational Study

**DOI:** 10.1155/2015/251485

**Published:** 2015-05-19

**Authors:** Grigorios Rombopoulos, Magdalini Hatzikou, Athanasios Athanasiadis, Moyses Elisaf

**Affiliations:** ^1^Novartis Hellas S.A., 12th klm National Road 1, Metamorfosis, 14451 Athens, Greece; ^2^Foundation for Economic and Industrial Research (IOBE), 11 Tsami Karatatsi Street, 11742 Athens, Greece; ^3^University Hospital of Ioannina, Stavros Niarchos Avenue, 45500 Ioannina, Greece

## Abstract

*Objective*. To evaluate the differences in treatment compliance with vildagliptin/metformin fixed-dose versus free-dose combination therapy in patients with type 2 diabetes mellitus (T2DM) in Greece. *Design*. Adult patients with T2DM, inadequately controlled with metformin monotherapy, (850 mg bid), participated in this 24-week, multicenter, observational study. Patients were enrolled in two cohorts: vildagliptin/metformin fixed-dose combination (group A) and vildagliptin metformin free-dose combination (group B). *Results*. 659 patients were enrolled, 360 were male, with mean BMI 30.1, mean T2DM duration 59.6 months, and mean HbA1c at baseline 8%; 366 patients were assigned to group A and 293 to group B; data for 3 patients was missing. In group A, 98.9% of patients were compliant with their treatment compared to 84.6% of group B. The odds ratio for compliance in group A versus B was (OR) 18.9 (95% CI: 6.2, 57.7; *P* < 0.001). In group A mean HbA1c decreased from 8.1% at baseline to 6.9% (*P* < 0.001) at the study end and from 7.9% to 6.8% (*P* < 0.001) in group B. *Conclusions*. Patients in group A were more compliant than patients in group B. These results are in accordance with international literature suggesting that fixed-dose combination therapies lead to increased compliance to treatment.

## 1. Introduction

Type 2 diabetes mellitus (T2DM) is a chronic, progressive disease. As glycemic control deteriorates over time, treatment intensification with the addition of multiple oral antihyperglycemic agents is often required in patients inadequately controlled with monotherapy [1]. Polypharmacy and complexity of the treatment regimens are associated with poor adherence to treatment, which in turn is associated with inadequate glycemic control [2–4]. On the other hand, the use of a fixed-dose combination of agents with complementary mechanisms of action is associated with improved patient compliance and adherence to treatment, as well as better glycemic control [5, 6].

Vildagliptin is a potent and selective oral dipeptidyl peptidase-4 inhibitor that improves glycemic control in patients with T2DM by increasing both the *α*-cell and *β*-cell responsiveness to glucose [7, 8]. In numerous clinical trials, combination therapy with vildagliptin and metformin has demonstrated a better efficacy and safety profile with good gastrointestinal tolerability than high-dose metformin monotherapy [9, 10]. A single-pill combination of vildagliptin/metformin has been approved in the European Union and across many countries in the world for the treatment of patients with T2DM inadequately controlled with metformin alone [11]. In the present study, we evaluated the differences in the treatment compliance with vildagliptin/metformin fixed-dose combination and vildagliptin (50 mg bid) added to metformin (free-dose combination) therapy in patients with T2DM in Greece.

## 2. Materials and Methods

This was a 24-week, multicenter, observational study. Patients aged >18 years with T2DM and inadequate glycemic control with metformin monotherapy (850 mg bid) were eligible to participate in the study. Patients were enrolled in two cohorts on 1 : 1 ratio, according to everyday clinical practice: those receiving either vildagliptin/metformin fixed-dose combination pill (hereafter referred to as the fixed-dose combination group) or vildagliptin (50 mg bid) added to metformin (850 mg bid) (hereafter referred to as the free-dose combination group).

Patients with a history of type 1 diabetes, end stage renal disease, undergoing hemodialysis, congestive heart failure, and pregnant or lactating women were excluded from the study. In order to assess the treatment compliance, investigators were asked to complete a compliance questionnaire by interviewing patients both at the baseline (visit 1) and final visit 3 (24 weeks after baseline) (Table 1). Patients were considered compliant if they did not miss any drug dose or no more than 2 doses per week, received the correct dosage of the medication, and did not interrupt their treatment. Treatment compliance was assessed from the compliance questionnaire, and the difference in compliance between the treatment groups was reported. In addition to the questionnaire, investigator collected clinical, demographic, and relevant medical history data including comorbidities and complications. At the baseline visit, each patient was given a diary to record their medication intake on a daily basis. The patient was asked to return this diary to the physician at the final visit.

The study was designed and conducted in accordance with the applicable local regulations and with the ethical principles laid down in the Declaration of Helsinki. A written, informed consent was requested from each patient before enrollment in the study.

### 2.1. Efficacy and Safety Assessments

The primary objective was to compare the percentage of patients compliant with their prescribed therapy. Secondary objectives of the study were to assess the changes in the levels of HbA1c from the baseline until the end of the study (day 0 to 6 months after) and to assess the safety and tolerability profile of vildagliptin.

### 2.2. Statistical Analysis

Assuming 60% of the patients on fixed-dose combination therapy were compliant and a difference in the treatment groups of 12%, 320 patients per treatment group were required to provide 90% power at a significance level of 5%. The primary variable, difference in compliance between the two treatment groups, was assessed using a multiple binary logistic regression model and adjusted for age, sex, comorbidities, concomitant medications, duration of T2DM, whether patients remembered the names of their medications for T2DM, difficulties in ingestion, and clinical laboratory test results.

The odds ratio (OR) with 95% confidence intervals (CI) was also calculated from the multiple binary logistic regression analysis. Change in HbA1c from baseline to end of study was analyzed using an analysis of covariance model (AN.CO.VA.). All adverse events (AEs) and serious adverse events (SAEs) were recorded and monitored, along with their severity and relationship to the study drug.

## 3. Results

Patient demographics and baseline characteristics were generally comparable between the two treatment groups (Table 2). Of the 659 patients enrolled, 366 (55.5%) were assigned to the fixed-dose combination group and 293 (44.5%) to the free-dose combination group; data for 3 patients were missing. Overall, 54.4% of patients were men, mean age was 61.9 years, mean body mass index (BMI) was 30.1 kg/m^2^, and mean baseline HbA1c was 8.0%. About 9% of patients were taking other concomitant medications and 16% of patients had comorbidities, of which 70% of patients had hypertension, 59% had dyslipidemia, and 12% had ischemic heart disease. Overall, 92.6% of patients were compliant with their prescribed therapy according to the definition of compliance used in this study.

**Figure 1:**
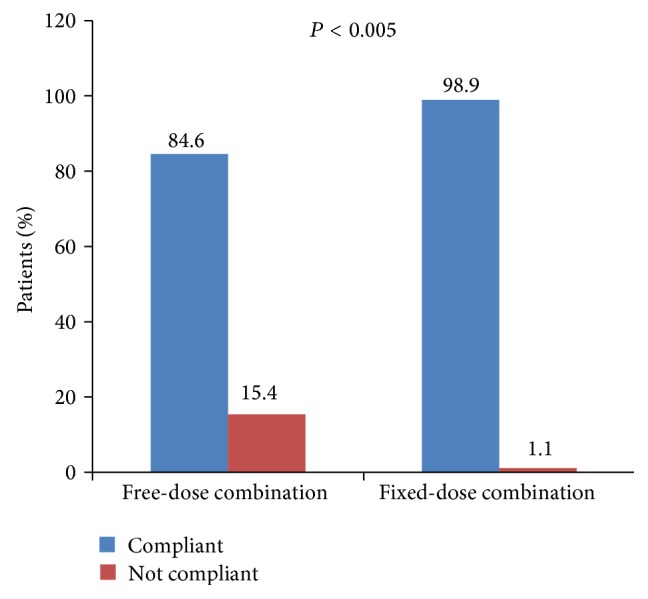
Percentage of patients compliant to treatment (logarithmic regression model, chi-square test).

The percentage of patients compliant with treatment in the fixed-dose combination group was 98.9% compared with 84.6% in the free-dose combination group before adjusting for confounding factors (Figure 1). The OR for compliance in the fixed-dose combination group versus the free-dose combination group was 18.9 (95% CI: 6.2, 57.7; *P* < 0.001) after adjusting for confounding factors. Patients who remembered the names of their medications were five times more likely to comply with their treatment than patients who did not remember the names of their medications. Patients who experienced difficulty in swallowing their medications were 31.3 times less likely to comply with their treatment compared with patients who did not experience any difficulty in swallowing their medications. The model was also tested for goodness of fit to the data of the study using the Hosmer and Lemeshow that proved that the model had a good fit to the study data; *P* value = 0.619 (Table 3).

**Figure 2:**
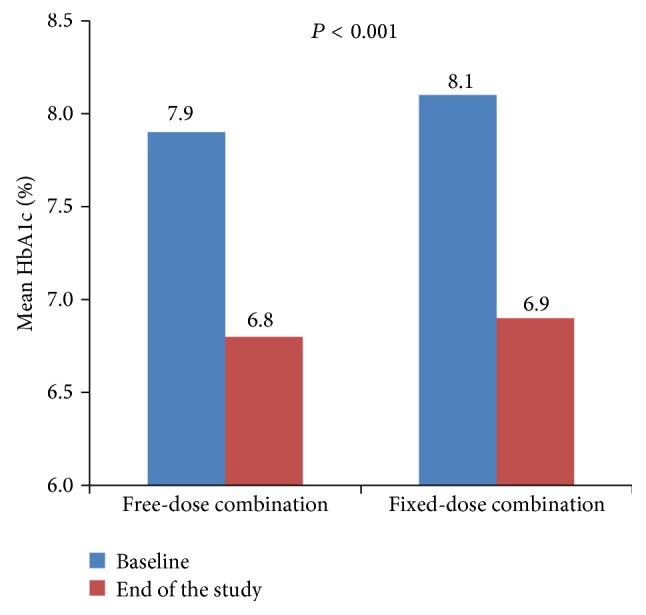
Mean HbA1c (%) at baseline and at end of study (logarithmic regression model, chi-square test).

The mean HbA1c decreased from a baseline of 8.1% to 6.9% in the fixed-dose combination group and from 7.9% to 6.8% in the free-dose combination group; the change was statistically significant from baseline to study end in both groups but not between groups (Figure 2). No serious AEs were reported during the study.

## 4. Discussion

Management of T2DM is complex due to multiple factors such as competing comorbidities, resistance to pharmacotherapy, reluctance to increase the dosage and/or the number of medications, low socioeconomic or educational status, and lack of adherence to lifestyle modifications [12]. All the above factors lead to poor treatment compliance. One practical way to enhance compliance in patients with multiple comorbidities and receiving concomitant medications is to simplify the treatment regimen with fixed-dose combinations. Results from meta-analysis of clinical trials showed that fixed-dose combinations reduce the risk of noncompliance and improve compliance with treatment compared with free-dose combination regimens [13, 14].

As treatment compliance may influence the overall glycemic control as well as progression of the disease, findings from this study may prove to be useful when assessing treatment strategies for diabetes mellitus. In the present observational study, more number of patients in the fixed-dose combination group were found to be compliant to the treatment (OR 18.9, 95% CI: 6.2, 57.7; *P* < 0.001) compared with the free-dose combination group. This is consistent with the findings from a meta-analysis of seven studies that reported 10% to 13% higher treatment adherence with fixed-dose combination of medications than with free-dose combinations [5]. In this study, patients who did not remember the names of their medications and those who experienced difficulty in swallowing their medications were less likely to comply with their treatment, suggesting that simple names for medications and pill size could help in improving the compliance with medication. The mean HbA1c decreased from a baseline of 8.1 to 6.9% in the fixed-dose combination group and from 7.9% to 6.8% in the free-dose combination group. The observed HbA1c drop in the present study is consistent with the results reported from a large clinical trial (−0.9 ± 0.1%) which assessed the efficacy and safety of vildagliptin add-on to metformin [15]. Of note, although there were differences with respect to treatment compliance between the fixed-dose and free-dose combinations, these did not result in a difference in efficacy. The results from the present study showed that the combination of two oral antihyperglycemic agents with complementary mechanisms of action offers benefits of consistent glycemic control and helps to improve medication compliance. In addition, there were no new safety signals observed with either fixed-dose or free-dose combinations of vildagliptin and metformin which was generally consistent with the previously reported tolerability profile of vildagliptin as add-on therapy to metformin [8].

The present study has certain limitations that need to be considered while interpreting the results. Only a few patients completed the diaries on a daily basis which resulted in inadequate data for additional analysis and, further, the 6-month follow-up period might be considered a short duration for the measurement of compliance and its effect on efficacy. Moreover, it should be added that the method assessing compliance (interview) is not as accurate as the pill count method or the microprocessor method.

In conclusion, patients on vildagliptin/metformin fixed-dose combination were more compliant with their treatment when compared with patients on free-dose combination. Taking into account that T2DM is a chronic disease, it is important to emphasize that its management should be a part of a health policy plan, and priority should be given to therapies with proven effectiveness and safety as well as fixed-dose combinations that improve patients' compliance.

## Figures and Tables

**Table 1:** Compliance questionnaire.

Variable	
Does the therapy affect the daily activities of the patient?	In acute degree
	In some degree
	No

Taking their medication at the same time every day?	Yes
	No

Does the patient have difficulties in swallowing the medication?	Yes
	No

How important do you consider that the therapy is in order to treat the disease?	Very important
	Important
	Of some importance

Have they missed any dose of the treatment?	Today
	Yesterday
	Last week
	Last 2 weeks
	Last month
	Not one dose

Percentage of medication received last month.	Mean
	SD

Does the patient remember the commercial names of the medications?	Yes
	No

Total number of daily tablets for the treatment of T2DM.	Mean
	SD

How often do they forget to take their treatment for T2DM.	Never/almost never
	1-2 times a month
	1 time in a week
	>1 time in a week
	Almost every day

Compliance.	Yes
	No

**Table 2:** Patient baseline and demographic characteristics.

	Free-dose combination group *N* = 293	Fixed-dose combination group *N* = 366	Total *N* = 659
Age, years	62.0 (9.6)	61.9 (8.7)	61.9 (9.1)
60–75, *n* (%)	141 (48.1)	192 (52.5)	334 (50.5)
>75, *n* (%)	23 (7.8)	16 (4.4)	39 (5.9)
Gender, men, *n* (%)	152 (51.9)	205 (56.0)	360 (54.4)
Body mass index, kg/m^2^	29.6 (3.9)	30.4 (4.04)	30.1 (4.0)
Duration of T2DM, months	55.4 (51.1)	62.4 (55.2)	59.6 (54.2)
Mean HbA1c, %	7.9 (0.8)	8.1 (0.9)	8.0 (0.8)
Comorbidity, yes, *n* (%)	54 (18.4)	49 (13.4)	104 (15.7)
Concomitant treatments, yes, *n* (%)	28 (9.6)	31 (8.5)	60 (9.1)

T2DM, type 2 diabetes mellitus; data are presented as mean (SD) unless otherwise specified.

**Table 3:** Log regression model on compliance and confounding factors.

Variables	*B*	*P* value	Exp(*B*)	95% CI for Exp(*B*)	
				Lower	Upper
Treatment (fixed versus free combination)	2,938	0.001	18,887	6,178	57,738
Medication recall visit 3	1,609	0.001	4,997	2,432	10,264
Swallowing difficulties visit 3	−2,574	0.001	0,076	0,032	0,184
Constant	3,488	0.220	32,728		
